# Increased ω6-Containing Phospholipids and Primary ω6 Oxidation Products in the Brain Tissue of Rats on an ω3-Deficient Diet

**DOI:** 10.1371/journal.pone.0164326

**Published:** 2016-10-27

**Authors:** Paul H. Axelsen, Robert C. Murphy, Miki Igarashi, Stanley I. Rapoport

**Affiliations:** 1 Departments of Pharmacology, Biochemistry and Biophysics, and Medicine, University of Pennsylvania School of Medicine, Philadelphia, PA, 19104–6084, United States of America; 2 Department of Pharmacology, Mail Stop 8303, University of Colorado at Denver Health Sciences Center, Aurora, CO, 80045–0511, United States of America; 3 Brain Physiology and Metabolism Section, National Institute on Aging, National Institutes of Health, Bethesda, MD, 20892, United States of America; Universidade de Sao Paulo Instituto de Quimica, BRAZIL

## Abstract

Polyunsaturated fatty acyl (PUFA) chains in both the ω3 and ω6 series are essential for normal animal brain development, and cannot be interconverted to compensate for a dietary deficiency of one or the other. Paradoxically, a dietary ω3-PUFA deficiency leads to the accumulation of docosapentaenoate (DPA, 22:5ω6), an ω6-PUFA chain that is normally scarce in the brain. We applied a high-precision LC/MS method to characterize the distribution of DPA chains across phospholipid headgroup classes, the fatty acyl chains with which they were paired, and the extent to which they were oxidatively damaged in the cortical brain of rats on an ω3-deficient diet. Results indicate that dietary ω3-PUFA deficiency markedly increased the concentrations of phospholipids with DPA chains across all headgroup subclasses, including plasmalogen species. The concentrations of phospholipids containing docosahexaenoate chains (22:6ω3) decreased 20–25%, while the concentrations of phospholipids containing arachidonate chains (20:4ω6) did not change significantly. Although DPA chains are more saturated than DHA chains, a larger fraction of DPA chains were monohydroxylated, particularly among diacyl-phosphatidylethanolamines and plasmalogen phosphatidylethanolamines, suggesting that they were disproportionately subjected to oxidative stress. Differences in the pathological significance of ω3 and ω6 oxidation products suggest that greater oxidative damage among the ω6 PUFAs that increase in response to dietary ω3 deficiency may have pathological significance in Alzheimer’s disease.

## Introduction

Arachidonic acid (ARA, 20:4ω6) and docosahexaenoic acid (DHA, 22:6ω3) are the most abundant polyunsaturated fatty acids (PUFAs) in mammalian brain [1–4]. They are involved in many cellular processes, including the regulation of membrane fluidity, cell signaling, and gene transcription, while their metabolites are involved in intercellular communication, inflammation, and various neuropathological processes [2–5].

ARA and DHA are deemed essential fatty acids because animal tissues have only a limited ability to synthesize them from precursors, and dietary intake is required for normal brain development [6,7]. To the extent that the synthesis of ARA and DHA is possible in animals, it occurs by elongation of the functionalized end of shorter PUFAs and the subsequent action of Δ5 and Δ6 desaturases. Thus, α-linolenic acid (α-LNA, 18:3ω3) may be elongated and desaturated to form eicosapentaenoic acid (EPA, 20:5ω3) and DHA, while linoleic acid (LA, 18:2ω6) and γ-linolenic acid, (γ-LNA, 18:3ω6) may be elongated and desaturated to form dihomo-γ-linolenic acid (DGLA, 20:3ω6), ARA, and docosapentaenoic acid (DPA, 22:5ω6). However, animal cells cannot alter the saturation of 18-carbon fatty acid chains beyond the C9,10 bond, so that ω3 and ω6 PUFAs cannot be interconverted regardless of chain length.

The inability to interconvert ω3 and ω6 PUFAs causes relatively uncommon PUFA species to accumulate in the brain when PUFAs are deficient in the diet [8–11]. With dietary ω3-PUFA deprivation, an increase in DPA is observed, while ω6-PUFA deprivation causes an increase in EPA. Within the brain, these conversions can occur in cerebral astrocytes and vascular endothelium [12–14]. In the case of ω3-PUFA deprivation, however, the activities of liver elongases and desaturases are upregulated, so that much of the DPA accumulated in the brain may have originated in the liver [9,10,15]. The net result is that the brain accumulates PUFA chains of the requisite length, even if they do not have the normal degree of unsaturation.

Prior studies of PUFA accumulation under conditions of dietary ω3 deprivation have characterized PUFA chain content by GC/MS after saponification and derivatization [10,16] or by GC/MS combined with limited ESI-MS/MS analysis [17]. LC/MS techniques can identify and quantify large numbers of individual PL species, which is not possible with GC/MS [18]. In this study, we employed LC/MS to examine the effect of dietary ω3-PUFA deprivation on the concentration of PUFA-containing PL species and some of their oxidation products in rat brain. In young rats on a diet that was nominally free of ω3-PUFAs for 15 weeks, the concentrations of DPA-containing PLs increased dramatically in all six major headgroup subclasses, particularly among PE plasmalogens. The concentrations of phospholipids containing ARA were unchanged or slightly increased, while those containing DHA were significantly decreased. Dietary ω3-PUFA deprivation also caused increases in PE species containing oxidized forms of DPA. Our results add precision and detail to previously reported shifts in brain PUFA content due to ω3-deprivation, demonstrating that the diet-induced increase in ω6-containing phospholipid species occurs across all individual species including plasmalogens within each headgroup subclass, and suggesting that the increased ω6-containing PE species are subject to a greater degree of oxidative stress.

## Materials and Methods

### Reagents

Neat arachidonic acid, docosapentaenoic acid, and docosahexaenoic acid were obtained from NuChek Prep (Elysian, MN). *d8*-arachidonic acid and *d5*-docosahexaenoic acid were purchased from Cayman Chemicals (Ann Arbor, MI). 17:0a/20:4a-PC, 17:0a/20:4a-PE, 17:0a/20:4a-PG, 17:0a/20:4a-PI, 17:0a/20:4a-PS, 17:0a/20:4a-PA, 21:0a/22:6a-PC, 21:0a/22:6a-PE, 21:0a/22:6a-PG, 21:0a/22:6a-PI, 21:0a/22:6a-PS, and 21:0a/22:6a-PA in methanol were purchased from Avanti Polar Lipids (Alabaster, AB). Pentafluorobenzyl bromide (PFB) and N,N-diisopropylethylamine (DIEA) were obtained from Sigma-Aldrich (St. Louis, MO).

### Animals and diets

The dietary and experimental protocol was approved by the Animal Care and Use Committee of the Eunice Kennedy Schriver National Institute of Child Health and Human Development and followed the National Institutes of Health Guide for the Care and Use of Laboratory Animals (NIH Publication 80–23). Fischer-344 (CDF) male rat pups (18 days old) and their surrogate mothers were purchased from Charles River Laboratories (Portage, MI) and housed in an animal facility with regulated temperature and humidity and a 12 h light/12 h dark cycle. The pups were allowed to nurse until 21 days old. Lactating rats had free access to water and to rodent chow formulation NIH-31 18–4, which contained 4% crude fat (w/w) (Zeigler Bros., Gardners, PA) and whose fatty acid composition has been reported [19]. α-LNA, EPA, and DHA (all ω3) contributed 5.1, 2.0, and 2.3% of total fatty acids, respectively, whereas LA and ARA (both ω6) contributed 47.9% and 0.02%, respectively. After weaning, the pups were divided randomly into two diet groups: one received a diet with nominally adequate amounts of ω3 PUFAs, while the other received a diet nominally deficient in ω3 PUFAs for 15 weeks. Both groups had free access to food and water, and their food was replaced every 2 or 3 days. Body weight was recorded once a week.

The ω3 adequate and ω3 deficient diets were prepared by Dyets, Inc. (Bethlehem, PA) with compositions based on the AIN-93G formulation [19] as previously described [9,20]. Each diet contained 10% fat. The adequate diet contained flaxseed oil but the deficient diet did not. The fatty acids (μmol/g food, percentage of total fatty acid, and percentage of food energy) in the diets were determined as previously described [10]. The adequate diet contained 7.8 μmol/g α-LNA (4.6% total fatty acid), a minimum level for diet ω3 PUFA adequacy in rodents [9,16,21]. The deficient diet contained 0.25 mmol/g α-LNA (0.2% total fatty acid). Other ω3 PUFAs were absent in both diets. Both contained 40 μmol/g diet LA (23–24% of total fatty acid), 110 μmol/g saturated fatty acid (65–69% of total), and 10 μmol/g monounsaturated fatty acid (5–6% of total).

### Sample processing

At the end of the 15 week dietary intervention, each animal was euthanized by an overdose of sodium pentobarbital (100 mg/kg i.v.), and its head was immediately subjected to 5.5 kW focused-beam high-energy microwave irradiation for 4.8 s (Model S6F; Cober Electronics, Stamford, CT). The brain was removed and confirmed visually to be browned; if not browned, it was discarded. Samples ranging in mass from 2.0–5.0 mg were removed from the parietal cortex while frozen. One sample was taken from each animal, yielding a total of 6 samples from animals on an ω3-adequate diet, and 6 samples from animals on an ω3-deficient diet. Frozen tissue samples were transferred to high-recovery clear borosilicate glass autosampler vials for weighing (9512S, Microsolv Technology Corp, Eatontown, NJ) and extracted using a modified Bligh-Dyer procedure following the addition of 17:0a- and 21:0a-containing internal standards as previously described [18]. Precautions were taken to protect both synthetic standards and samples from air-oxidation including grinding the samples under liquid nitrogen, the use of dicholoromethane instead of choloroform to avoid exposure of lipids to phosgene, and a minimum of solution transfers. Antioxidants were not included since so-called antioxidants may also have pro-oxidant activity [22]. The samples were stored at -80°C for up to 1 month before processing. Other than a brief period during weighing, tissue samples continuously remained frozen in liquid nitrogen (-196°C), dry ice (-78°C), or freezer (-80°C) until extracted.

### Mass spectrometry

Four multiple reaction monitoring (MRM) analytical methods were developed for an ABI 4000 QTrap mass spectrometer (ABSciex, Toronto, Canada). One method monitored a set of collision-induced mass transitions in the negative ion mode corresponding to the production of ARA anions (*m/z* 303.2) from various phospholipid [M−H]^−^ and [M+OAc^−^]^−^ anions, and the 17:0 heptadacanoate anions (*m/z* 269.2) derived from *sn1* chains in the ARA-containing standards. A second method monitored the corresponding mass transitions for DHA anions (*m/z* 327.2) and 21:0 heneicosanoate anions (*m/z* 327.3). A third method monitored the corresponding mass transitions for DPA anions (*m/z* 329.2) and 21:0 heneicosanoate anions. A fourth method was devised for monitoring selected PE species containing mono-hydroxy PUFA chains. A complete list of mass transitions is provided as Supporting Information (S1 Data). Each method involved a normal phase chromatographic separation and a 10 μl of each extract were injected onto a 4.6 x 250 mm silica column (Rx-SIL, Agilent), through which solvents were pumped at 1 ml/min. Solvent A was 30 parts hexanes and 40 parts isopropanol; solvent B was 30 parts hexanes, 40 parts isopropanol, and 7 parts 11 mM ammonium acetate in water. Solvent B was increased linearly from 30% to 98% over 10 min, and was held at 98% for 15 min. The column was re-equilibrated with 30% solvent B for at least 5 min before another sample was injected. Column effluent was directed into a high precision flow splitter (Analytical Scientific Instruments, El Sobrante, CA) with precisely 25% of the effluent directed into the standard electrospray ionization (ESI) source for LC/MS/MS analysis, and 75% collected in fractions for GC/MS analysis.

Each method had 4 time periods during which different transitions were monitored, as indicated in Fig 2 of reference 18. Phosphatidylglycerol (PG) species were monitored in period 1 between 7.0 and 9.5 min; phosphatidylinositol (PI) and phosphatidylethanolamine (PE) species were monitored in period 2 between 8.5 and 11.5 min; phosphatidic acid (PA) and phosphatidylserine (PS) species were monitored in period 3 between 11.5 and 13.5 min; and phosphatidylcholine (PC) species were monitored in period 4 between 18 and 19 min.

For each transition, the dwell time was 100 msec, the source voltage was -4500 V, the declustering potential was -75 V, the collision energy was –40 eV, the collision gas was set to “medium”, the resolution for both the first and third quadrupoles were set to “unit”, and a drying gas at 300°C was applied to the spray. Transition peaks were integrated using Analyst 1.4.2 software, although all integrations were visually reviewed, and many were adjusted manually.

Each extract was injected once for each of the four MRM methods. The methods for ARA, DHA, and DPA anions each yielded 28 measurements (27 phospholipid species plus transitions arising from the internal standards) for each of 6 phospholipid headgroup subclasses, or 6 x 28 = 168 individual measurements (table of transitions provided in S1 Data). The column effluent was collected in 5 fractions as indicated in Fig 2 of reference 18. The ARA, DPA, and DHA contents of each fraction were determined by GC/MS.

Fractions for GC/MS analysis were saponified with 1M NH_4_OH, mixed with *d8*-ARA and *d5*-DHA internal standards, esterified with PFB in DIEA, and extracted into isooctane as described previously [23]. GC/MS was performed with a ZB-1 polydimethylsiloxanecapillary gas chromatograph column (30 m x0.2 mm inner diameter x 0.25 μm film thickness; Phenomenex, Torrance, CA) attached to a ThermoFinnigan (San Jose, CA) Trace DSQ mass spectrometer. The injector temperature was maintained at 230°C, and the transfer line was kept at 290°C. The mass spectrometric experiments were performed in negative CI mode (70 eV) with a source temperature of 200°C. Helium was used as the carrier gas, with a constant flow rate of 0.8 ml/min. A standard curve for converting the internal standard signals to concentration was prepared with solutions of neat ARA and DHA in methanol that were derivatized in the same manner.

### Data Analysis

The integrated signal for each monitored mass transition was corrected for ^13^C-content and processed as described in Results. Quantitative sensitivity varied with the inherent difficulty of detecting lipid species in some headgroup subclasses, and the amounts of these headgroup subclasses present in the brain. In addition, the results were subject to several assumptions that render them somewhat approximate. One assumption was that ARA and DHA chains always occupied the *sn2* position, as in the synthetic standards. However, lipids may have these chains in the *sn1* position, they may have both an ARA and a DHA chain, and they may even have two ARA or two DHA chains. Precise corrections for these uncertainties are not available. No attempt was made to correct for differences in ionization efficiency due to differences in mass, or due to differences between ether-linked and acyl-linked *sn1* chains.

For PG and PC lipids, the conversion of MRM signals to quantitative results was performed by:

PLn=ra,n(FAx,wra,sum⋅0.75⋅(10350)) (1)

where *PL*_*n*_is the average concentration of an individual phospholipid species *n*,

$r_{a,n}=\frac{1}{6}\sum_{i=1}^{6}\left(c_{a,n,i}^{\prime }/c_{a,s,i}^{\prime }\right)$(2) (6 samples from each diet averaged separately)

$FA_{x,w}=\frac{1}{12}\sum_{i=1}^{12}\left(FA_{x,i}/w_{i}\right)$(3) (averaged over all 12 samples)

$r_{a,sum}=\frac{1}{12}\sum_{i=1}^{12}\sum_{n=1}^{28}r_{a,n,i}$(4) (the sum of 28 transitions averaged over all 12 samples)

$c_{a,n,i}^{\prime }$ is the signal recorded for phospholipid species *n* in headgroup subclass *a* for sample *i*, and $c_{a,s,i}^{\prime }$, is the signal recorded for an internal standard in the same headgroup subclass and sample. Primes indicates that the signal has been corrected for ^13^C content, *FA* is the fatty acyl content of the collected column effluent fraction *x* for sample *i* in nmoles, *w* is the mass of tissue sample *i* in grams, 0.75 is the fraction of the column effluent that was collected by the fraction collector, and 10/350 is the portion of the tissue extract that was injected onto the column. It should be noted that the term in parentheses in Eq 1 is constant for all phospholipid species within headgroup subclass. Therefore, the relative values of *PL*_*n*_ for different sample sets depend only on the count ratio averages (*r*_*a*,*n*_) and are independent of each other.

For PI/PE and PA/PS lipids, data reduction requires information about the relative sensitivity of the MRM method for these headgroup subclasses, given by the ratios *s*_*ab*_ of signals recorded from an equimolar mixture of a lipid in headgroup subclass *a*, and a lipid in headgroup subclass *b* (provided in table 3 of ref. 18). Assuming that the sum of the signals recorded for all lipid species in a headgroup subclass, corrected for the sensitivity of the MRM method for lipids in that headgroup subclass, is proportional to the molar concentration of the lipids in that headgroup subclass, it follows that a fraction containing lipids in two different headgroup subclasses may be divided according to:

$$FA_{a}=FA_{x,w}\left(\frac{c_{a,tot}}{c_{a,tot}+s_{ab}c_{b,tot}}\right)$$

and

$$FA_{b}=FA_{x,w}\left(\frac{s_{ab}c_{b,tot}}{c_{a,tot}+s_{ab}c_{b,tot}}\right)$$

where

$$c_{a,tot}=\sum_{n=1}^{27}c_{a,n}^{\prime }$$

and

$$c_{b,tot}=\sum_{n=1}^{27}c_{b,n}^{\prime }.$$

The concentration of an individual phospholipid is obtained by substituting either *FA*_*a*_ or *FA*_*b*_ for *FA*_*x*,*w*_ in Eq 1.

Results are reported as means and standard deviations for 6 separate tissue samples. The mean values for *r*_*a*,*n*_ were determined as in Eq 2, and scaled by the factor in parenthesis in Eq 1. The uncertainties indicated in the bar graphs are the standard deviation among *r*_*a*,*n*_ determinations from Eq 2, scaled by the factor in parenthesis in Eq 1. Because the scale factor in Eq 1 is the same for animals in both diet groups, the uncertainty in this scale factor was not propagated through to the final results. The strengths and weaknesses of this analytical approach have been discussed in detail [18], and additional comments are offered below in the discussion of results.

Phospholipids containing monohydroxy-PUFAs were detected by adding 16 daltons to both the parent ion and the fragment ion masses for a selected transition, and they were quantified by calculating *r*_*a*,*n*_ according to Eq 2 with *a* = PE. The internal standard was 17:0a/20:4a-PE in each case, and no made no attempt to distinguish among PUFAs hydroxylated at various positions. Therefore, results are expressed simply as the ratio *r*_*a*,*n*_(*deficient*)/*r*_*a*,*n*_(*adequate*). The uncertainty estimates were calculated as the square-root of the sum of the variances. Statistical tests were performed using the two-sample two-tailed T-test as implemented in Excel with P-value thresholds as indicated with the results.

## Results

Results of fatty acid analysis by GC/MS of five different phospholipid classes separated by normal phase chromatography are listed in Table 1. The ω3-deficient diet caused no significant change in the ARA content of any fraction. In contrast, the DPA content increased by 80% while the DHA content decreased by 17% on the ω3-deficient diet.

**Table 1:** ARA, DPA, and DHA content in the brain tissue of animals fed diets with adequate or deficient amounts of ω3-PUFAs. Fractions correspond to the five normal phase LC fractions described previously [18]. GC/MS determinations were made as described in methods. LC/MS/MS results are the sum of all data in Figs 1–3. Uncertainties for the five fractions are standard deviations of 6 measurements. Uncertainties for the fraction totals were calculated according to the variance sum law. All units are nmoles/gm tissue wet weight.

Fraction	LipidSubclass	ARA		DPA		DHA	
		ω3 adequate	ω3 deficient	ω3 adequate	ω3 deficient	ω3 adequate	ω3 deficient
0	Neutral	108 ± 43	146 ± 42	5 ± 2	7 ± 3	32 ± 10	24 ± 6
1	PG	89 ± 40	89 ± 36	1 ± 0	2 ± 0	15 ± 8	12 ± 3
2	PI,PE	4,419 ± 235	4,931 ± 1,034	409 ± 146	706 ± 208	5,858 ± 998	4,820 ± 784
3	PA,PS	410 ± 53	319 ± 48	73 ± 25	171 ± 64	1,035 ± 189	902 ± 218
4	PC	2,110 ± 202	2,316 ± 507	92 ± 37	158 ± 45	1,115 ± 200	910 ± 160
GC/MS Total, fractions 1–4	PL	7,027 ± 317	7,654 ± 1,153	576 ± 153	1,037 ± 222*	8,024 ± 1,036	6,643 ± 830*
LC/MS/MS Total, fractions 1–4	PL	7,238 ± 314	7,445 ± 577	202 ± 19	1,410 ± 95*	8,251 ± 773	6,426 ± 550*

*P < 0.005 compared to corresponding ω3-adequate samples.

**Fig 1:**
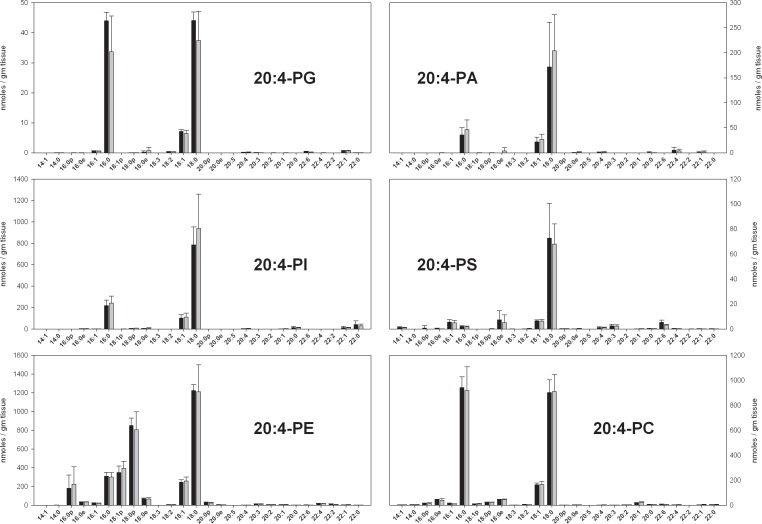
The concentrations of ARA-containing phospholipid species in the parietal cortex of animals fed a diet with adequate amounts of ω3 PUFAs (black bars) and a diet deficient in ω3 PUFAs (gray bars) in nmoles / gm tissue. Each result is the mean of 6 different brain extracts, and the error bars represent standard deviations. The absence of a bar for an sn1 chain indicates that the corresponding MRM transition was monitored, but no signal detected. There were no statistically significant differences between groups. See text for information about the nature of quantitative uncertainty in these data.

ARA-containing PL in rat brain were qualitatively similar to the previously described ARA-containing PL in mouse brain (Fig 1) [18]. The largest quantitative differences were a 30% decrease in PI species, a 50% decrease in PS species, and 3-fold increase in PA species in rats relative to mice. It should be noted that PS and PA species are relatively small components of the ARA-containing phospholipidome, and that the larger components in microwaved rat brain (this work) and flash-frozen mouse brain (ref 18) are quantitatively similar. No significant differences in individual ARA-containing PL, or in total ARA-containing PL (Table 1) were apparent between ω3-adequate and ω3-deficient diets.

**Fig 2:**
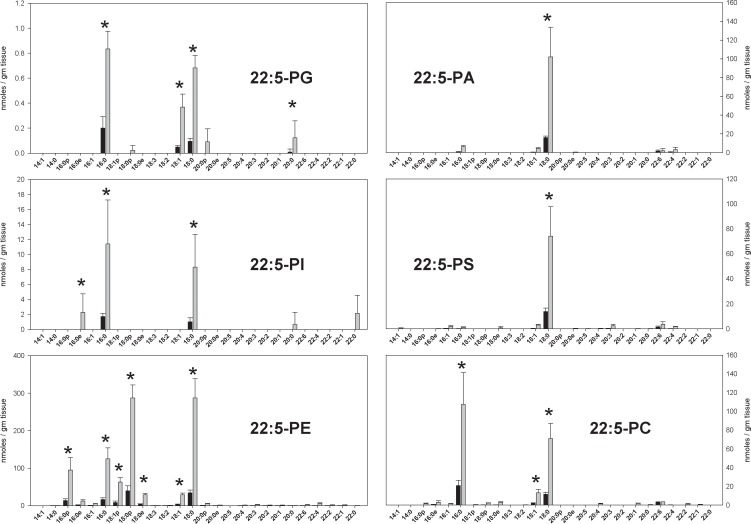
The concentrations of DPA-containing phospholipid species in the parietal cortex of animals fed a diet with adequate amounts of ω3 PUFAs (black bars) and a diet deficient in ω3 PUFAs (gray bars) in nmoles / gm tissue. Each result is the mean of 6 different brain extracts, and the error bars represent standard deviations. The absence of a bar for an sn1 chain indicates that the corresponding MRM transition was monitored, but no signal detected. An asterisk (*) indicates results where differences between diet groups were significant at the p < 0.001 level after Benjamini & Hochberg correction for false discovery rate. See text for information about the nature of quantitative uncertainty in these data.

DPA-containing PL levels in all headgroup subclasses were markedly increased in rats on the ω3-deficient diet (Fig 2). Virtually every PL species present in measurable quantities had increased, in some cases 10-fold. In rats on the ω3-adequate diet, the absolute quantities of all DPA-containing PL species were small compared to the corresponding ARA-containing species. On the ω3-deficient diet, PL quantities in the PG, PI, and PC headgroup subclasses remained small relative to the corresponding ARA-containing species. However, DPA-containing PL species in the PE, PA, and PS subclasses contributed substantially to the total ω6-PUFA content of the brain tissue, constituting roughly 25%, 50% and 100% (i.e. equal to) the corresponding ARA-containing PL species in those subclasses.

**Fig 3:**
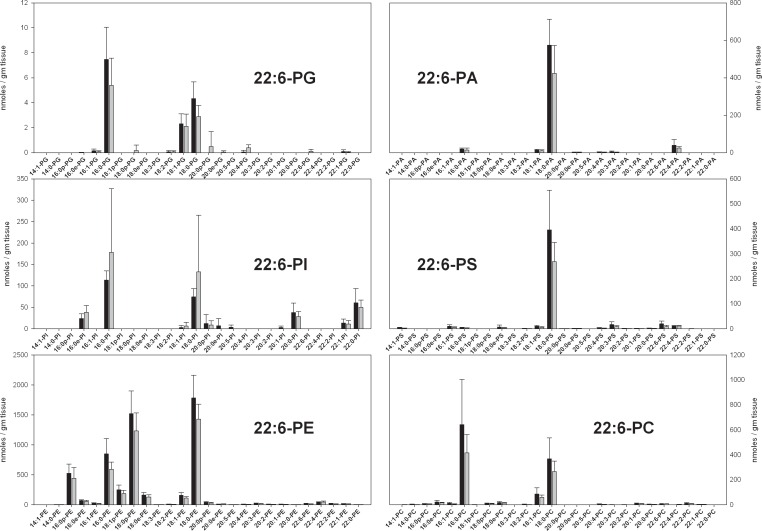
The concentrations of DHA-containing phospholipid species in the parietal cortex of animals fed a diet with adequate amounts of ω3 PUFAs (black bars) and a diet deficient in ω3 PUFAs (gray bars) in nmoles / gm tissue. Each result is the mean of 6 different brain extracts, and the error bars represent standard deviations. The absence of a bar for an sn1 chain indicates that the corresponding MRM transition was monitored, but no signal detected. Despite a clear trend for lower values in the ω3-deficient groups, there were no statistically significant differences. See text for information about the nature of quantitative uncertainty in these data.

DHA-containing PL species in rat brain were qualitatively and quantitatively similar to the previously described ARA-containing PL in mouse brain (Fig 3). The largest differences were a 50% decrease in PI species, and slightly higher amounts of PS and PA species in rats. As with ARA-containing species, PS and PA are relatively small components of the DHA-containing phospholipidome, and the majority components from microwaved rat brain and flash-frozen mouse brain are quantitatively similar. There were no statistically significant differences in DHA-containing phospholipid species between ω3-adequate and ω3-deficient diets, despite a 22% decrease in DHA-containing PL in animals on ω3-deficient diets (Table 1).

**Fig 4:**
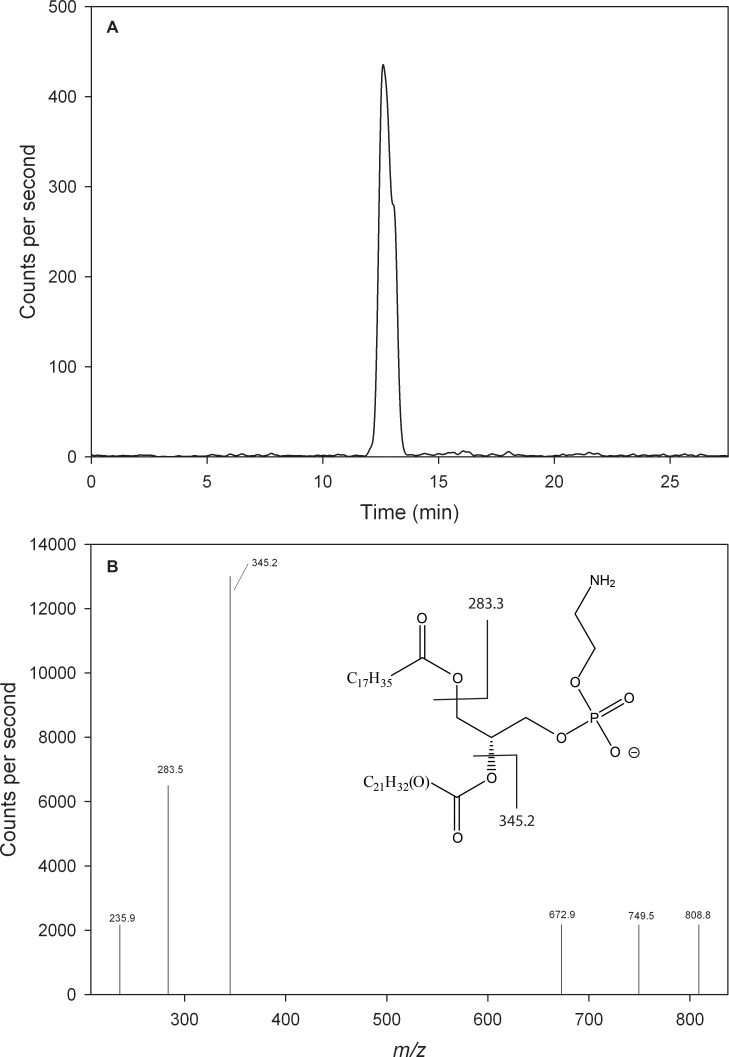
Identification of 18:0/22:5(OH)-PE by LC/MS/MS. (a) Single reaction monitoring chromatogram of the 808.8 → 345.2 transition, with solvent flow reduced to 850 μl/min. 18:0/22:5(+O)-PE eluted 0.3 min later than 18:0/22:5-PE, consistent with the slightly greater polarity of the oxidized species. (b) Product ions produced by the *m/z* 808.8 parent ion at 12.6 min. Due to the low concentrations of the parent species (a maximum of only 400 transitions/sec observed in panel a), this spectrum was collected with the Q1 resolution set to “unit”, while the Q3 resolution was set to “open”. The position of the oxygen on the 22:5 chain is unknown, and possibly variable. The identity of the *m/z* 672.9 and 749.5 product ions is not known.

Because the most abundant DPA-containing species were in the PE subclass, an MRM-LC/MS/MS method was developed to detect oxidized forms of the eight most abundant PUFA-containing PE species in animals on DHA-deficient diets. In each case, the parent oxidized form and the fragment ion being monitored were 16 Da greater than the corresponding transition for the unoxidized form. A representative chromatogram for the transition in which 18:0/22:5(+O)-PE yields a 22:5(+O) fragment (m/z 808.8 → 345.2) is shown in Fig 4A. A product ion spectrum for the m/z 808.8 ion yielded fragments correponding to the 18:0 (*m/z* 283.3) and 22:5(+O) (*m/z* 345.2) chains (Fig 4B).

**Fig 5:**
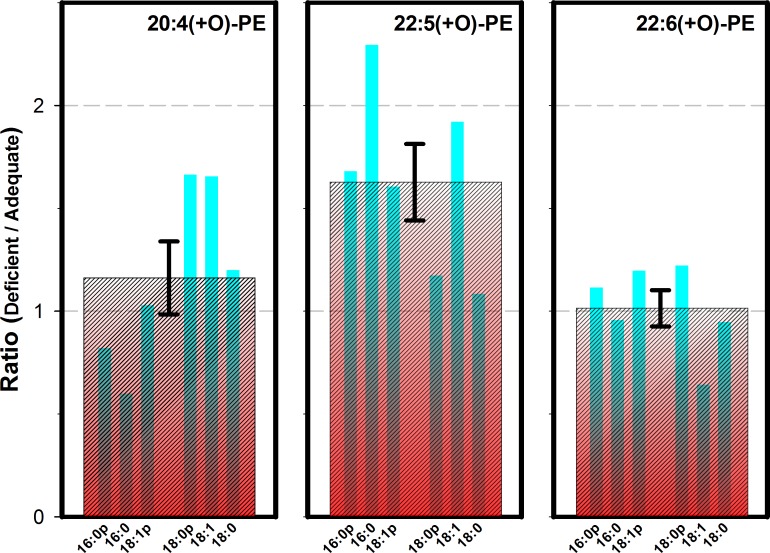
The ratio of +O addition (oxidation) for selected PE species in the parietal cortex of animals fed the diet deficient in ω3 PUFAs, relative to the diet with adequate amounts of ω3 PUFAs. The three panels represent PE species containing either oxidized (a) ARA, (b) DPA, or (c) DHA. Thin blue bars in each panel represent results for the *sn*-1 chains indicated, which correspond to the 6 most abundant PE species in Figs 1–3. The wide red cross-hatched bar in each panel is the average ratio for all 6 species in aggregate, with a standard error of the mean indicated. By 2-tailed T test of unpaired samples with unequal variances, the ratio for 22:5(+O)-PE species in aggregate (1.63) differs from the ratio for 20:4(+O)-PE species (1.16) at P ≤ 0.05, and from the ratio for 22:6(+O)-PE species (1.01) at P ≤ 0.01.

Absolute quantitative determinations of oxidized species were not possible by MRM-LC/MS/MS because reference and co-eluting internal standards were not available, and because species oxidized at different positions could not be distinguished by these methods. The same considerations also complicate GC/MS analysis of the saponified fatty acid chains. However, a precise ratio of the fraction oxidized of various species in ω3-deficient and ω3-adequate diets can be determined by MRM-LC/MS/MS (Fig 5). Results indicate that DPA chains in PE species tended to be oxidized to a greater degree in animals fed a ω3-deficient diet, in one case by a factor of 2.3. The results for oxidized ARA-containing PE species were mixed, while results for most oxidized DHA-containing PE species were largely unchanged. The *sn2*-chains in phospholipids with acyl-linked *sn1*-chains and plasmalogens were oxidized to a similar extent, but no oxidized *sn2*-chains were observed in conjunction with ether-linked *sn1*-chains (data not shown). Compared in aggregate, the ω3 deficient/adequate ratio for oxidized DPA chains was significantly higher than for oxidized ARA or DHA chains.

## Discussion

These results show that a dietary ω3-PUFA deficiency for 15 weeks following weaning, causes marked increases in the concentration of DPA-containing PLs in the rat brain across all six major headgroup classes. On a ω3-adequate diet, DPA-containing PLs comprised less than 3% of ARA-containing or DHA-containing PLs in brain (Table 1). After 15 weeks on the ω3-deficient diet, DPA-containing PLs increased to 19% of ARA-containing PL levels and 22% of DHA-containing PL levels. The increases were greatest for 18:0/22:5-PE and 18:0p/22:5-PE, but evident in all headgroup subclasses (Fig 2). The overall increase in DPA-containing PLs (~1200 nmoles/gm) corresponded to approximately 2/3 of the overall decrease in DHA-containing PLs (~1800 nmoles/gm, Table 1). These general relationships were previously detected using GC/FID [10,16] techniques, but the current results add to previously reported observations by (a) demonstrating that the increases in DPA-containing PLs occur in all headgroup subclasses, (b) providing more precise results than previously available from GC/MS or GC/FID, and (c) revealing that the diet-induced DPA chains are disproportionately susceptible to oxidative damage. As discussed below, the displacement of ω3 chains by ω6-DPA in a fatty acyl chain pool that is disproportionately susceptible to oxidative damage, has implications for the development of amyloid plaques in Alzheimer’s disease [24–30].

Dietary ω3 deficiency increases the hepatic activity of Δ5 and Δ6 desaturases, and elongases 2 and 5 [9,10,15] which, in turn, facilitate the synthesis of DHA from 20-carbon ω3-PUFA precursors, namely EPA and α-LNA, via the ω3 DPA isomer (22:5ω3) [7,31].Under conditions of dietary deficiency, the production of DPA in the liver is increased due to the action of these enzymes on 20-carbon ω6 precursors LA and ARA [9,10,15]. It is not yet known whether increased brain DPA content is due to an increase in the activity of uptake mechanisms, an increased availability of DPA due to hepatic production, or a lack of competition from dietary DHA for imperfectly selective DHA uptake mechanisms. However, with graded dietary deprivation, the increased brain DPA appears to follow increased availability of unesterified circulating DPA, which in turn follows its increased synthesis and secretion by the liver [32]. The negative mode MRM-LC/MS approach used in this work has been previously used to quantify PL species containing ARA and DHA in mouse brain [18]. A limitation of this approach for this study is that it cannot distinguish between ARA and 20:4ω3, or between DPA and 22:5ω3. However, both 20:4ω3 and 22:5ω3 are precursors in the biosynthesis of DHA, and we would expect both of them to be depleted on a ω3-PUFA deficient diet.

ARA has multiple prominent pharmacological roles in signaling, and is a precursor for numerous bioactive eicosanoids. DHA also has clear and essential roles in human physiology. Its derivatives include resolvins and neuroprotectins, which have roles in inflammation, gene transcription and other important processes [33,34]. For example, DHA and its 15-LOX product, 10,17S-docosatriene, decrease hippocampal NF-κB and COX-2 gene expression in other models [35]. Oxidative degradation of ARA gives rise to 20-carbon isoprostanes [36,37] and the corresponding 22-carbon derivatives of DHA are known as neuroprostanes [38,39]. Both isoprostanes and neuroprostanes are produced non-enzymatically, and have no known biological function, but are sometimes used as surrogate measures of oxidative stress. Likewise, DPA may be enzymatically oxidized by lipoxygenases [40], and 12-LOX can convert DPA to anti-inflammatory oxylipins [41] and resolvins [42], although DPA levels in RBC membranes are also correlated with elevated inflammatory markers [43]. Elevated brain DPA concentrations in rats fed a 0.2% α-LNA diet are accompanied by increased DPA turnover in brain phospholipids, which may be related to the increased activity of Ca^2+^-dependent cytosolic phospholipase A_2_ in the deprived rats [44]. The loss of one double bond from DHA to DPA alters the distribution of chain densities along the bilayer normal, and most likely the activity of enzymes intimately related to the phospholipid membrane [45], possibly accounting for the effect of DPA on visual signal transduction [46]. Effects of this nature may account for behavioral abnormalities noted in the ω3-PUFA deficient rat [16]

The results in Fig 5 show that dietary ω3-PUFA deficiency leads to relatively higher levels of oxidatively damaged DPA in PE lipids, in addition to increased DPA levels. The oxidation products identified in this work are most likely monohydroxy-PUFAs, since the parent ions yielded the expected product ions, they eluted as single peaks, their retention times were slightly longer than the corresponding non-hydroxylated species in this normal-phase system (Fig 4), and they are the most abundant type of PUFA oxidation product found in some models of oxidative damage [47]. However, isobaric oxidation products such as epoxides have not been ruled out, and chains that are oxidized at different positions are not separated. The increase in monohydroxy-DPA seems more likely due to an increase in chemically-mediated (e.g. free-radical-initiated) oxidative stress, rather than increased enzymatic (e.g. lipoxygenase) activity, because there were no corresponding increases evident in monohydroxy-ARA or–DHA, the natural substrates for such enzymes. If chemically-mediated, however, DPA must be in a cellular pool that is somehow more susceptible to oxidative stress than ARA or DHA.

It is in this context that the consequences of dietary ω3-PUFA deficiency may provide insight into the pathogenesis of Alzheimer’s disease. At least one ω6-PUFA oxidation product is amyloidogenic while its corresponding ω3-PUFA oxidation product is not [27], suggesting that dietary ω3-PUFA deficiency may render brain tissue more vulnerable to amyloidogenic effects of oxidative stress by increasing the production of ω6-PUFA oxidation products [41,48]. Conversely, dietary DHA supplementation alleviates the histopathological signs of Alzheimer’s disease in mice, and in neuronal cell cultures [33,49–55]. Therefore, the development of amyloid in brain may depend to some extent on the degree to which ω6-PUFAs such as DPA displace ω3-PUFAs such as DHA in a pool that is vulnerable to oxidative damage.

## Supporting Information

S1 Data Tables of MRM transitions monitored for phospholipid species containing ARA, DPA, and DHA. (PDF)

## Abbreviations

ESI: electrospray ionization
MRM: multiple reaction monitoring
MS: mass spectrometry
MS/MS: tandem mass spectrometry
LC/MS: liquid chromatography coupled to mass spectrometry
GC/MS: gas chromatography coupled to mass spectrometry
GC/FID: gas chromatography coupled to flame ionization detection
CAD: collision induced dissociation
PUFA: polyunsaturated fatty acid
TG: triglyceride
PL: phospholipid
PG: phosphatidylglycerol
PI: phosphatidylinositol
PE: phosphatidylethanolamine
PA: phosphatidic acid
PS: phosphatidylserine
PC: phosphatidylcholine
ARA: arachidonic acid or arachidonate, 20:4ω6
DHA: docosahenaenoic acid or docosahenaenoate, 22:6ω3
LA: linoleic acid, 18:2ω6
α-LNA: α-linolenic acid, 18:3ω3
γ-LNA: γ-linolenic acid, 18:3ω6
EPA: eicosapentaenoic acid, 20:5ω3
DPA: docosapentaenoic acid, 22:5ω6
