# Supplementary material for: Increased ω6-Containing Phospholipids and Primary ω6 Oxidation Products in the Brain Tissue of Rats on an ω3-Deficient Diet
Source: PLoS One. 2016 Oct 27;11(10):e0164326. doi: 10.1371/journal.pone.0164326 (PMC5082804; doi:10.1371/journal.pone.0164326)
Supplement: S1 Data — (PDF) [file pone.0164326.s001.pdf]

Supplementary table 1a. MRM transitions monitored for ARA-containing phospholipids

14:1/20:4-PG	739.5 / 303.3	14:1/20:4-PI	827.5 / 303.3	14:1/20:4-PE	708.5 / 303.3	14:1/20:4-PA	665.4 / 303.3	14:1/20:4-PS	752.5 / 303.3	14:1/20:4-PC	810.5 / 303.3
14:0/20:4-PG	741.5 / 303.3	14:0/20:4-PI	829.5 / 303.3	14:0/20:4-PE	710.5 / 303.3	14:0/20:4-PA	667.4 / 303.3	14:0/20:4-PS	754.5 / 303.3	14:0/20:4-PC	812.5 / 303.3
16:0p/20:4-PG	753.5 / 303.3	16:0p/20:4-PI	841.5 / 303.3	16:0p/20:4-PE	722.5 / 303.3	16:0p/20:4-PA	679.5 / 303.3	16:0p/20:4-PS	766.5 / 303.3	16:0p/20:4-PC	824.6 / 303.3
16:0e/20:4-PG	755.5 / 303.3	16:0e/20:4-PI	843.5 / 303.3	16:0e/20:4-PE	724.5 / 303.3	16:0e/20:4-PA	681.5 / 303.3	16:0e/20:4-PS	768.5 / 303.3	16:0e/20:4-PC	826.6 / 303.3
16:1/20:4-PG	767.5 / 303.3	16:1/20:4-PI	855.5 / 303.3	16:1/20:4-PE	736.5 / 303.3	16:1/20:4-PA	693.5 / 303.3	16:1/20:4-PS	780.5 / 303.3	16:1/20:4-PC	838.5 / 303.3
16:0/20:4-PG	769.5 / 303.3	16:0/20:4-PI	857.5 / 303.3	16:0/20:4-PE	738.5 / 303.3	16:0/20:4-PA	695.5 / 303.3	16:0/20:4-PS	782.5 / 303.3	16:0/20:4-PC	840.6 / 303.3
18:1p/20:4-PG	779.5 / 303.3	18:1p/20:4-PI	867.5 / 303.3	18:1p/20:4-PE	748.5 / 303.3	18:1p/20:4-PA	705.5 / 303.3	18:1p/20:4-PS	792.5 / 303.3	18:1p/20:4-PC	850.6 / 303.3
18:0p/20:4-PG	781.5 / 303.3	18:0p/20:4-PI	869.5 / 303.3	18:0p/20:4-PE	750.5 / 303.3	18:0p/20:4-PA	707.5 / 303.3	18:0p/20:4-PS	794.5 / 303.3	18:0p/20:4-PC	852.6 / 303.3
18:0e/20:4-PG	783.5 / 303.3	18:0e/20:4-PI	871.5 / 303.3	18:0e/20:4-PE	752.5 / 303.3	18:0e/20:4-PA	709.5 / 303.3	18:0e/20:4-PS	796.5 / 303.3	18:0e/20:4-PC	854.6 / 303.3
17:0/20:4-PG	783.5 / 269.2	17:0/20:4-PI	871.5 / 269.2	17:0/20:4-PE	752.5 / 269.2	17:0/20:4-PA	709.5 / 269.2	17:0/20:4-PS	796.5 / 269.2	17:0/20:4-PC	854.6 / 269.2
18:3/20:4-PG	791.5 / 303.3	18:3/20:4-PI	879.5 / 303.3	18:3/20:4-PE	760.5 / 303.3	18:3/20:4-PA	717.5 / 303.3	18:3/20:4-PS	804.5 / 303.3	18:3/20:4-PC	862.5 / 303.3
18:2/20:4-PG	793.5 / 303.3	18:2/20:4-PI	881.5 / 303.3	18:2/20:4-PE	762.5 / 303.3	18:2/20:4-PA	719.5 / 303.3	18:2/20:4-PS	806.5 / 303.3	18:2/20:4-PC	864.6 / 303.3
18:1/20:4-PG	795.5 / 303.3	18:1/20:4-PI	883.5 / 303.3	18:1/20:4-PE	764.5 / 303.3	18:1/20:4-PA	721.5 / 303.3	18:1/20:4-PS	808.5 / 303.3	18:1/20:4-PC	866.6 / 303.3
18:0/20:4-PG	797.5 / 303.3	18:0/20:4-PI	885.5 / 303.3	18:0/20:4-PE	766.5 / 303.3	18:0/20:4-PA	723.5 / 303.3	18:0/20:4-PS	810.5 / 303.3	18:0/20:4-PC	868.6 / 303.3
20:0p/20:4-PG	809.6 / 303.3	20:0p/20:4-PI	897.5 / 303.3	20:0p/20:4-PE	778.6 / 303.3	20:0p/20:4-PA	735.5 / 303.3	20:0p/20:4-PS	822.6 / 303.3	20:0p/20:4-PC	880.6 / 303.3
20:0e/20:4-PG	811.6 / 303.3	20:0e/20:4-PI	899.5 / 303.3	20:0e/20:4-PE	780.6 / 303.3	20:0e/20:4-PA	737.6 / 303.3	20:0e/20:4-PS	824.3 / 303.3	20:0e/20:4-PC	882.6 / 303.3
20:5/20:4-PG	815.6 / 303.3	20:5/20:4-PI	903.5 / 303.3	20:5/20:4-PE	784.6 / 303.3	20:5/20:4-PA	741.5 / 303.3	20:5/20:4-PS	828.3 / 303.3	20:5/20:4-PC	886.5 / 303.3
20:4/20:4-PG	817.6 / 303.3	20:4/20:4-PI	905.5 / 303.3	20:4/20:4-PE	786.5 / 303.3	20:4/20:4-PA	743.5 / 303.3	20:4/20:4-PS	830.5 / 303.3	20:4/20:4-PC	888.6 / 303.3
20:3/20:4-PG	819.6 / 303.3	20:3/20:4-PI	907.5 / 303.3	20:3/20:4-PE	788.5 / 303.3	20:3/20:4-PA	745.5 / 303.3	20:3/20:4-PS	832.5 / 303.3	20:3/20:4-PC	890.6 / 303.3
20:2/20:4-PG	821.6 / 303.3	20:2/20:4-PI	909.6 / 303.3	20:2/20:4-PE	790.5 / 303.3	20:2/20:4-PA	747.5 / 303.3	20:2/20:4-PS	834.5 / 303.3	20:2/20:4-PC	892.6 / 303.3
20:1/20:4-PG	823.6 / 303.3	20:1/20:4-PI	911.6 / 303.3	20:1/20:4-PE	792.6 / 303.3	20:1/20:4-PA	749.5 / 303.3	20:1/20:4-PS	836.6 / 303.3	20:1/20:4-PC	894.6 / 303.3
20:0/20:4-PG	825.6 / 303.3	20:0/20:4-PI	913.6 / 303.3	20:0/20:4-PE	794.6 / 303.3	20:0/20:4-PA	751.5 / 303.3	20:0/20:4-PS	838.6 / 303.3	20:0/20:4-PC	896.6 / 303.3
22:6/20:4-PG	841.6 / 303.3	22:6/20:4-PI	929.6 / 303.3	22:6/20:4-PE	810.5 / 303.3	22:6/20:4-PA	767.5 / 303.3	22:6/20:4-PS	854.5 / 303.3	22:6/20:4-PC	912.6 / 303.3
22:4/20:4-PG	845.6 / 303.3	22:4/20:4-PI	933.6 / 303.3	22:4/20:4-PE	814.5 / 303.3	22:4/20:4-PA	771.5 / 303.3	22:4/20:4-PS	858.5 / 303.3	22:4/20:4-PC	916.6 / 303.3
22:2/20:4-PG	849.6 / 303.3	22:2/20:4-PI	937.6 / 303.3	22:2/20:4-PE	818.6 / 303.3	22:2/20:4-PA	775.5 / 303.3	22:2/20:4-PS	862.6 / 303.3	22:2/20:4-PC	920.6 / 303.3
22:1/20:4-PG	851.6 / 303.3	22:1/20:4-PI	939.6 / 303.3	22:1/20:4-PE	820.6 / 303.3	22:1/20:4-PA	777.6 / 303.3	22:1/20:4-PS	864.6 / 303.3	22:1/20:4-PC	922.6 / 303.3
22:0/20:4-PG	853.6 / 303.3	22:0/20:4-PI	941.6 / 303.3	22:0/20:4-PE	822.6 / 303.3	22:0/20:4-PA	779.6 / 303.3	22:0/20:4-PS	866.6 / 303.3	22:0/20:4-PC	924.7 / 303.3

Supplementary table 1b. MRM transitions monitored for DHA-containing phospholipids

14:1/22:6-PG	763.5 / 327.2	14:1/22:6-PI	851.5 / 327.2	14:1/22:6-PE	732.5 / 327.2	14:1/22:6-PA	689.4 / 327.2	14:1/22:6-PS	776.5 / 327.2	14:1/22:6-PC	834.5 / 327.2
14:0/22:6-PG	765.5 / 327.2	14:0/22:6-PI	853.5 / 327.2	14:0/22:6-PE	734.5 / 327.2	14:0/22:6-PA	691.4 / 327.2	14:0/22:6-PS	778.5 / 327.2	14:0/22:6-PC	836.5 / 327.2
16:0p/22:6-PG	777.5 / 327.2	16:0p/22:6-PI	865.5 / 327.2	16:0p/22:6-PE	746.5 / 327.2	16:0p/22:6-PA	703.5 / 327.2	16:0p/22:6-PS	790.5 / 327.2	16:0p/22:6-PC	848.6 / 327.2
16:0e/22:6-PG	779.5 / 327.2	16:0e/22:6-PI	867.5 / 327.2	16:0e/22:6-PE	748.5 / 327.2	16:0e/22:6-PA	705.5 / 327.2	16:0e/22:6-PS	792.5 / 327.2	16:0e/22:6-PC	850.6 / 327.2
16:1/22:6-PG	791.5 / 327.2	16:1/22:6-PI	879.5 / 327.2	16:1/22:6-PE	760.5 / 327.2	16:1/22:6-PA	717.5 / 327.2	16:1/22:6-PS	804.5 / 327.2	16:1/22:6-PC	862.5 / 327.2
16:0/22:6-PG	793.5 / 327.2	16:0/22:6-PI	881.5 / 327.2	16:0/22:6-PE	762.5 / 327.2	16:0/22:6-PA	719.5 / 327.2	16:0/22:6-PS	806.5 / 327.2	16:0/22:6-PC	864.6 / 327.2
18:1p/22:6-PG	803.5 / 327.2	18:1p/22:6-PI	891.5 / 327.2	18:1p/22:6-PE	772.5 / 327.2	18:1p/22:6-PA	729.5 / 327.2	18:1p/22:6-PS	816.5 / 327.2	18:1p/22:6-PC	874.6 / 327.2
18:0p/22:6-PG	805.5 / 327.2	18:0p/22:6-PI	893.5 / 327.2	18:0p/22:6-PE	774.6 / 327.2	18:0p/22:6-PA	731.5 / 327.2	18:0p/22:6-PS	818.5 / 327.2	18:0p/22:6-PC	876.6 / 327.2
18:0e/22:6-PG	807.5 / 327.2	18:0e/22:6-PI	895.6 / 327.2	18:0e/22:6-PE	776.6 / 327.2	18:0e/22:6-PA	733.5 / 327.2	18:0e/22:6-PS	820.6 / 327.2	18:0e/22:6-PC	878.6 / 327.2
18:3/22:6-PG	815.5 / 327.2	18:3/22:6-PI	903.5 / 327.2	18:3/22:6-PE	784.5 / 327.2	18:3/22:6-PA	741.5 / 327.2	18:3/22:6-PS	828.5 / 327.2	18:3/22:6-PC	886.5 / 327.2
18:2/22:6-PG	817.5 / 327.2	18:2/22:6-PI	905.5 / 327.2	18:2/22:6-PE	786.5 / 327.2	18:2/22:6-PA	743.5 / 327.2	18:2/22:6-PS	830.5 / 327.2	18:2/22:6-PC	888.6 / 327.2
18:1/22:6-PG	819.5 / 327.2	18:1/22:6-PI	907.5 / 327.2	18:1/22:6-PE	788.5 / 327.2	18:1/22:6-PA	745.5 / 327.2	18:1/22:6-PS	832.5 / 327.2	18:1/22:6-PC	890.6 / 327.2
18:0/22:6-PG	821.5 / 327.2	18:0/22:6-PI	909.5 / 327.2	18:0/22:6-PE	790.5 / 327.2	18:0/22:6-PA	747.5 / 327.2	18:0/22:6-PS	834.5 / 327.2	18:0/22:6-PC	892.6 / 327.2
20:0p/22:6-PG	833.6 / 327.2	20:0p/22:6-PI	921.5 / 327.2	20:0p/22:6-PE	802.6 / 327.2	20:0p/22:6-PA	759.5 / 327.2	20:0p/22:6-PS	846.6 / 327.2	20:0p/22:6-PC	904.6 / 327.2
20:0e/22:6-PG	835.6 / 327.2	20:0e/22:6-PI	923.6 / 327.2	20:0e/22:6-PE	804.6 / 327.2	20:0e/22:6-PA	761.6 / 327.2	20:0e/22:6-PS	848.6 / 327.2	20:0e/22:6-PC	906.6 / 327.2
20:5/22:6-PG	839.6 / 327.2	20:5/22:6-PI	927.5 / 327.2	20:5/22:6-PE	808.5 / 327.2	20:5/22:6-PA	765.5 / 327.2	20:5/22:6-PS	852.5 / 327.2	20:5/22:6-PC	910.5 / 327.2
20:4/22:6-PG	841.6 / 327.2	20:4/22:6-PI	929.5 / 327.2	20:4/22:6-PE	810.5 / 327.2	20:4/22:6-PA	767.5 / 327.2	20:4/22:6-PS	854.5 / 327.2	20:4/22:6-PC	912.6 / 327.2
20:3/22:6-PG	843.6 / 327.2	20:3/22:6-PI	931.5 / 327.2	20:3/22:6-PE	812.5 / 327.2	20:3/22:6-PA	769.5 / 327.2	20:3/22:6-PS	856.5 / 327.2	20:3/22:6-PC	914.6 / 327.2
20:2/22:6-PG	845.6 / 327.2	20:2/22:6-PI	933.6 / 327.2	20:2/22:6-PE	814.5 / 327.2	20:2/22:6-PA	771.5 / 327.2	20:2/22:6-PS	858.5 / 327.2	20:2/22:6-PC	916.6 / 327.2
20:1/22:6-PG	847.6 / 327.2	20:1/22:6-PI	935.6 / 327.2	20:1/22:6-PE	816.6 / 327.2	20:1/22:6-PA	773.5 / 327.2	20:1/22:6-PS	860.6 / 327.2	20:1/22:6-PC	918.6 / 327.2
20:0/22:6-PG	849.6 / 327.2	20:0/22:6-PI	937.6 / 327.2	20:0/22:6-PE	818.6 / 327.2	20:0/22:6-PA	775.5 / 327.2	20:0/22:6-PS	862.6 / 327.2	20:0/22:6-PC	920.6 / 327.2
22:0e/22:6-PG	863.6 / 327.2	22:0e/22:6-PI	951.5 / 327.2	22:0e/22:6-PE	832.5 / 327.2	22:0e/22:6-PA	789.5 / 327.2	22:0e/22:6-PS	876.5 / 327.2	22:0e/22:6-PC	934.6 / 327.2
21:0/22:6-PG	863.6 / 325.2	21:0/22:6-PI	951.5 / 325.2	21:0/22:6-PE	832.5 / 325.2	21:0/22:6-PA	789.5 / 325.2	21:0/22:6-PS	876.5 / 325.2	21:0/22:6-PC	934.6 / 325.2
22:6/22:6-PG	865.6 / 327.2	22:6/22:6-PI	953.5 / 327.2	22:6/22:6-PE	834.5 / 327.2	22:6/22:6-PA	791.5 / 327.2	22:6/22:6-PS	878.5 / 327.2	22:6/22:6-PC	936.6 / 327.2
22:4/22:6-PG	869.6 / 327.2	22:4/22:6-PI	957.6 / 327.2	22:4/22:6-PE	838.5 / 327.2	22:4/22:6-PA	795.5 / 327.2	22:4/22:6-PS	882.5 / 327.2	22:4/22:6-PC	940.6 / 327.2
22:2/22:6-PG	873.6 / 327.2	22:2/22:6-PI	961.6 / 327.2	22:2/22:6-PE	842.6 / 327.2	22:2/22:6-PA	799.5 / 327.2	22:2/22:6-PS	886.6 / 327.2	22:2/22:6-PC	944.6 / 327.2
22:1/22:6-PG	875.6 / 327.2	22:1/22:6-PI	963.6 / 327.2	22:1/22:6-PE	844.6 / 327.2	22:1/22:6-PA	801.6 / 327.2	22:1/22:6-PS	888.6 / 327.2	22:1/22:6-PC	946.6 / 327.2
22:0/22:6-PG	877.6 / 327.2	22:0/22:6-PI	965.6 / 327.2	22:0/22:6-PE	846.6 / 327.2	22:0/22:6-PA	803.6 / 327.2	22:0/22:6-PS	890.6 / 327.2	22:0/22:6-PC	948.7 / 327.2

Supplementary table 1c. MRM transitions monitored for DPA-containing phospholipids

14:1/22:5-PG	765.5 / 329.2	14:1/22:5-PI	853.5 / 329.2	14:1/22:5-PE	734.5 / 329.2	14:1/22:5-PA	691.4 / 329.2	14:1/22:5-PS	778.5 / 329.2	14:1/22:5-PC	836.5 / 329.2
14:0/22:5-PG	767.5 / 329.2	14:0/22:5-PI	855.5 / 329.2	14:0/22:5-PE	736.5 / 329.2	14:0/22:5-PA	693.4 / 329.2	14:0/22:5-PS	780.5 / 329.2	14:0/22:5-PC	838.5 / 329.2
16:0p/22:5-PG	779.5 / 329.2	16:0p/22:5-PI	867.5 / 329.2	16:0p/22:5-PE	748.5 / 329.2	16:0p/22:5-PA	705.5 / 329.2	16:0p/22:5-PS	792.5 / 329.2	16:0p/22:5-PC	850.6 / 329.2
16:0e/22:5-PG	781.5 / 329.2	16:0e/22:5-PI	869.5 / 329.2	16:0e/22:5-PE	750.5 / 329.2	16:0e/22:5-PA	707.5 / 329.2	16:0e/22:5-PS	794.5 / 329.2	16:0e/22:5-PC	852.6 / 329.2
16:1/22:5-PG	793.5 / 329.2	16:1/22:5-PI	881.5 / 329.2	16:1/22:5-PE	762.5 / 329.2	16:1/22:5-PA	719.5 / 329.2	16:1/22:5-PS	806.5 / 329.2	16:1/22:5-PC	864.5 / 329.2
16:0/22:5-PG	795.5 / 329.2	16:0/22:5-PI	883.5 / 329.2	16:0/22:5-PE	764.5 / 329.2	16:0/22:5-PA	721.5 / 329.2	16:0/22:5-PS	808.5 / 329.2	16:0/22:5-PC	866.6 / 329.2
18:1p/22:5-PG	805.5 / 329.2	18:1p/22:5-PI	893.5 / 329.2	18:1p/22:5-PE	774.5 / 329.2	18:1p/22:5-PA	731.5 / 329.2	18:1p/22:5-PS	818.5 / 329.2	18:1p/22:5-PC	876.6 / 329.2
18:0p/22:5-PG	807.5 / 329.2	18:0p/22:5-PI	895.5 / 329.2	18:0p/22:5-PE	776.6 / 329.2	18:0p/22:5-PA	733.5 / 329.2	18:0p/22:5-PS	820.5 / 329.2	18:0p/22:5-PC	878.6 / 329.2
18:0e/22:5-PG	809.5 / 329.2	18:0e/22:5-PI	897.6 / 329.2	18:0e/22:5-PE	778.6 / 329.2	18:0e/22:5-PA	735.5 / 329.2	18:0e/22:5-PS	822.6 / 329.2	18:0e/22:5-PC	880.6 / 329.2
18:3/22:5-PG	817.5 / 329.2	18:3/22:5-PI	905.5 / 329.2	18:3/22:5-PE	786.5 / 329.2	18:3/22:5-PA	743.5 / 329.2	18:3/22:5-PS	830.5 / 329.2	18:3/22:5-PC	888.5 / 329.2
18:2/22:5-PG	819.5 / 329.2	18:2/22:5-PI	907.5 / 329.2	18:2/22:5-PE	788.5 / 329.2	18:2/22:5-PA	745.5 / 329.2	18:2/22:5-PS	832.5 / 329.2	18:2/22:5-PC	890.6 / 329.2
18:1/22:5-PG	821.5 / 329.2	18:1/22:5-PI	909.5 / 329.2	18:1/22:5-PE	790.5 / 329.2	18:1/22:5-PA	747.5 / 329.2	18:1/22:5-PS	834.5 / 329.2	18:1/22:5-PC	892.6 / 329.2
18:0/22:5-PG	823.5 / 329.2	18:0/22:5-PI	911.5 / 329.2	18:0/22:5-PE	792.5 / 329.2	18:0/22:5-PA	749.5 / 329.2	18:0/22:5-PS	836.5 / 329.2	18:0/22:5-PC	894.6 / 329.2
20:0p/22:5-PG	835.6 / 329.2	20:0p/22:5-PI	923.5 / 329.2	20:0p/22:5-PE	804.6 / 329.2	20:0p/22:5-PA	761.5 / 329.2	20:0p/22:5-PS	848.6 / 329.2	20:0p/22:5-PC	906.6 / 329.2
20:0e/22:5-PG	837.6 / 329.2	20:0e/22:5-PI	925.6 / 329.2	20:0e/22:5-PE	806.6 / 329.2	20:0e/22:5-PA	763.6 / 329.2	20:0e/22:5-PS	850.6 / 329.2	20:0e/22:5-PC	908.6 / 329.2
20:5/22:5-PG	841.6 / 329.2	20:5/22:5-PI	929.5 / 329.2	20:5/22:5-PE	810.5 / 329.2	20:5/22:5-PA	767.5 / 329.2	20:5/22:5-PS	854.5 / 329.2	20:5/22:5-PC	912.5 / 329.2
20:4/22:5-PG	843.6 / 329.2	20:4/22:5-PI	931.5 / 329.2	20:4/22:5-PE	812.5 / 329.2	20:4/22:5-PA	769.5 / 329.2	20:4/22:5-PS	856.5 / 329.2	20:4/22:5-PC	914.6 / 329.2
20:3/22:5-PG	845.6 / 329.2	20:3/22:5-PI	933.5 / 329.2	20:3/22:5-PE	814.5 / 329.2	20:3/22:5-PA	771.5 / 329.2	20:3/22:5-PS	858.5 / 329.2	20:3/22:5-PC	916.6 / 329.2
20:2/22:5-PG	847.6 / 329.2	20:2/22:5-PI	935.6 / 329.2	20:2/22:5-PE	816.5 / 329.2	20:2/22:5-PA	773.5 / 329.2	20:2/22:5-PS	860.5 / 329.2	20:2/22:5-PC	918.6 / 329.2
20:1/22:5-PG	849.6 / 329.2	20:1/22:5-PI	937.6 / 329.2	20:1/22:5-PE	818.6 / 329.2	20:1/22:5-PA	775.5 / 329.2	20:1/22:5-PS	862.6 / 329.2	20:1/22:5-PC	920.6 / 329.2
20:0/22:5-PG	851.6 / 329.2	20:0/22:5-PI	939.6 / 329.2	20:0/22:5-PE	820.6 / 329.2	20:0/22:5-PA	777.5 / 329.2	20:0/22:5-PS	864.6 / 329.2	20:0/22:5-PC	922.6 / 329.2
22:0e/22:5-PG	865.6 / 329.2	22:0e/22:5-PI	953.5 / 329.2	22:0e/22:5-PE	834.5 / 329.2	22:0e/22:5-PA	791.5 / 329.2	22:0e/22:5-PS	878.5 / 329.2	22:0e/22:5-PC	936.6 / 329.2
21:0/22:6-PG	863.6 / 325.2	21:0/22:6-PI	951.5 / 325.2	21:0/22:6-PE	832.5 / 325.2	21:0/22:6-PA	789.5 / 325.2	21:0/22:6-PS	876.5 / 325.2	21:0/22:6-PC	934.6 / 325.2
22:5/22:5-PG	867.6 / 329.2	22:5/22:5-PI	955.5 / 329.2	22:5/22:5-PE	836.5 / 329.2	22:5/22:5-PA	793.5 / 329.2	22:5/22:5-PS	880.5 / 329.2	22:5/22:5-PC	938.6 / 329.2
22:4/22:5-PG	871.6 / 329.2	22:4/22:5-PI	959.6 / 329.2	22:4/22:5-PE	840.5 / 329.2	22:4/22:5-PA	797.5 / 329.2	22:4/22:5-PS	884.5 / 329.2	22:4/22:5-PC	942.6 / 329.2
22:2/22:5-PG	875.6 / 329.2	22:2/22:5-PI	963.6 / 329.2	22:2/22:5-PE	844.6 / 329.2	22:2/22:5-PA	801.5 / 329.2	22:2/22:5-PS	888.6 / 329.2	22:2/22:5-PC	946.6 / 329.2
22:1/22:5-PG	877.6 / 329.2	22:1/22:5-PI	965.6 / 329.2	22:1/22:5-PE	846.6 / 329.2	22:1/22:5-PA	803.6 / 329.2	22:1/22:5-PS	890.6 / 329.2	22:1/22:5-PC	948.6 / 329.2
22:0/22:5-PG	879.6 / 329.2	22:0/22:5-PI	967.6 / 329.2	22:0/22:5-PE	848.6 / 329.2	22:0/22:5-PA	805.6 / 329.2	22:0/22:5-PS	892.6 / 329.2	22:0/22:5-PC	950.7 / 329.2
